# Regulation of Na^+^ channel inactivation by the DIII and DIV voltage-sensing domains

**DOI:** 10.1085/jgp.201611678

**Published:** 2017-03-06

**Authors:** Eric J. Hsu, Wandi Zhu, Angela R. Schubert, Taylor Voelker, Zoltan Varga, Jonathan R. Silva

**Affiliations:** 1 Department of Biomedical Engineering, Washington University in St. Louis, St. Louis, MO 63130; 2 MTA-DE-NAP B Ion Channel Structure-Function Research Group, Research Center for Molecular Medicine (RCMM), University of Debrecen, Debrecen H-4032, Hungary

## Abstract

Functional eukaryotic voltage-gated Na^+^ (Na_V_) channels comprise four domains (DI–DIV), each containing six membrane-spanning segments (S1–S6). Voltage sensing is accomplished by the first four membrane-spanning segments (S1–S4), which together form a voltage-sensing domain (VSD). A critical Na_V_ channel gating process, inactivation, has previously been linked to activation of the VSDs in DIII and DIV. Here, we probe this interaction by using voltage-clamp fluorometry to observe VSD kinetics in the presence of mutations at locations that have been shown to impair Na_V_ channel inactivation. These locations include the DIII–DIV linker, the DIII S4–S5 linker, and the DIV S4-S5 linker. Our results show that, within the 10-ms timeframe of fast inactivation, the DIV-VSD is the primary regulator of inactivation. However, after longer 100-ms pulses, the DIII–DIV linker slows DIII-VSD deactivation, and the rate of DIII deactivation correlates strongly with the rate of recovery from inactivation. Our results imply that, over the course of an action potential, DIV-VSDs regulate the onset of fast inactivation while DIII-VSDs determine its recovery.

## Abstract (precis)

Hsu et al. probe voltage-gated Na^+^ channels that are inactivation deficient with voltage-clamp fluorometry. They find that in the time domain of an action potential, the voltage-sensing domain (VSD) of domain IV regulates fast inactivation onset while the domain III VSD determines its recovery.

## Introduction

In neurons and myocytes, voltage-gated Na^+^ (Na_V_) channels initiate membrane excitation. Shortly thereafter, fast inactivation diminishes the Na^+^ current, which allows K^+^ efflux to return the membrane to its resting potential. Inactivation kinetics cannot be described by a single time constant, but instead require a collection of relaxations whose time domains span from milliseconds to minutes (Silva, 2014; Marom, 2016). After myocyte and neuron membranes return to their resting potential, the rate of recovery from Na_V_ channel inactivation determines the period during which they are refractory to excitation. In addition to the conduction velocity of excitation, the refractory period plays a key role in determining whether the myocardium is able to sustain deadly arrhythmic activity (Mines, 1913).

Much effort has been put into mapping inactivation to the eukaryotic Na_V_ channel structure. Functional Na^+^ channels are formed by monomers with four homologous domains (DI–DIV), each with six transmembrane-spanning segments (S1–S6). DI–DIV are each composed of a voltage-sensing domain (VSD) formed by S1–S4 and a pore domain that includes S5–S6. Within the VSD, a charged segment (S4) responds to changes in membrane potential to activate and deactivate the channel (Catterall et al., 2005; Ahern et al., 2016). Inactivation is associated with a hydrophobic amino acid sequence (Ile-Phe-Met-Thr) known as the IFM or IFMT motif that is located within the DIII–DIV linker (Vassilev et al., 1988; Stühmer et al., 1989; West et al., 1992). Inactivation is also significantly modulated by the S4–S5 linkers of DIII (Smith and Goldin, 1997) and DIV (McPhee et al., 1998), stretches of the DI-S6 and DIV-S6 segments (McPhee et al., 1994, 1995; Wang et al., 2003), and the C terminus (Deschênes et al., 2001).

Insight into the role that the VSDs play in modulating inactivation has been gained by tethering fluorophores to the extracellular S4 segments (Chanda and Bezanilla, 2002; Chanda et al., 2004), which allows correlation of changes in VSD conformation to ionic current kinetics, including inactivation (Cha et al., 1999; Silva and Goldstein, 2013a,b). Recent application of this method, known as voltage-clamp fluorometry (VCF), has shown that DIV-VSD conformation is tightly connected to the rapid components of inactivation in the rat skeletal muscle Na^+^ channels, rNa_V_1.4 (Capes et al., 2013). The DIII-VSD of rNa_V_1.4 has been shown to modulate channel opening (Muroi et al., 2010) and interact with pore-binding local anesthetics (Sheets and Hanck, 2003; Muroi and Chanda, 2009; Arcisio-Miranda et al., 2010), but its inactivation role is less well defined.

In the human cardiac Na_V_ channel, hNa_V_1.5, inactivation defects predispose patients to arrhythmia and sudden death. In long QT syndrome type 3 patients, hNa_V_1.5 inactivation is often impaired, and the increase in persistent Na^+^ current causes prolonged action potentials and triggered activity. In contrast, Brugada syndrome mutations often enhance hNa_V_1.5 inactivation, which reduces peak Na^+^ current and causes pro-arrhythmic conduction abnormalities (Silva and Silva, 2012; Havakuk and Viskin, 2016). 14 mutations within the DIII-VSD have been shown to cause SCN5A-linked channelopathies (UniProt Consortium, 2015), suggesting a prominent role for the DIII-VSD in regulating inactivation.

We have recently developed VCF protocols for hNa_V_1.5 and observed that the DIII-VSD was uniquely immobilized by prolonged inactivation-inducing pulses (Varga et al., 2015; Zhu et al., 2016). Here, we apply these methods to observe the VSD effects of inactivation-deficient mutations within the DIII–DIV linker, the DIII S4–S5 linker, and the DIV S4–S5 linker to discover how they interact to regulate Na_V_ channel inactivation.

## Materials and methods

### Molecular biology

Mutation primers were purchased from Sigma-Aldrich. All mutagenesis was performed using the QuikChange II Site-Directed Mutagenesis kit (Agilent Technologies). DNA plasmids were purified with Midiprep kits (MACHEREY-NAGEL), and the mMESSAGE mMACHINE T7 Transcription kit (Thermo Fisher Scientific) was used to synthesize capped mRNA.

### Cut-open VCF recordings

Cut-open oocyte VCF was used to record cardiac Na^+^ channel ionic currents and fluorescence from oocytes as previously described (Rudokas et al., 2014; Varga et al., 2015). cRNAs for the human β1 subunit (UniProtKB/Swiss-Prot accession no. Q07699) and α-subunit Na_V_1.5 (accession no. Q14524) were produced from the pBSTA and pMAX vectors (respectively) and injected at a 2:1 molar ratio (55 ng per cell total) into *Xenopus laevis* oocytes. Oocytes were incubated individually at 18°C for 4–6 d in ND93 solution (93 mM NaCl, 5 mM KCl, 1.8 mM CaCl_2_, 1 mM MgCl_2_, 5 mM HEPES, 2.5 mM Na-pyruvate, and 1% penicillin-streptomycin, pH 7.4). Cut-open oocyte recordings were performed using an amplifier (CA-1B; Dagan Corporation) coupled to an A/D converter (Digidata 1440; Molecular Devices) with Clampex and Clampfit software (v10; Molecular Devices) for acquisition and analysis. The temperature of the chambers was maintained at 19°C with a temperature controller (HCC-100A; Dagan Corporation). Internal solution consisted of 105 mM NMG-Mes, 10 mM Na-Mes, 20 mM HEPES, and 2 mM EGTA, pH 7.4. External solution consisted of 25 mM NMG-Mes, 90 mM Na-Mes, 20 mM HEPES, and 2 mM Ca-Mes_2_, pH 7.4.

Oocytes were labeled with 20 µM methanethiosulfonate-carboxytetramethylrhodamine (MTS-TAMRA; Santa Cruz Biotechnology, Inc.) in a depolarizing solution (110 mM KCl, 1.5 mM MgCl_2_, 0.8 mM CaCl_2_, 0.2 mM EDTA, and 10 mM HEPES, pH 7.1) on ice for 30–40 min. MTS-TAMRA stock solution was 10 mM in DMSO and stored at −20°C. Illumination was provided by a green, high-powered LED (Luminus, PT-121) controlled through a driver (LDPC-30-6-24VDC; Lumina Power) by the acquisition software to minimize photobleaching of the probe. The light was then focused into a liquid light guide with a 45°, 5-mm compound parabolic concentrator (Edmund Optics), and the guide was coupled to the microscope via a collimating adapter (EXFO). Fluorescence emission was captured by a 40× water-immersion objective with a numerical aperture of 0.8 (CFI Plan Fluor; Nikon) and quantified with a photodiode (PIN-040A; United Detector Technology) mounted on an XY axis manipulator (Thorlabs, Inc.) at the microscope epifluorescence port. The collected light was focused onto the photodiode active area using an achromatic doublet (Thorlabs, Inc.) with a focal distance of 25 mm, and the photodiode was attached to the integrating headstage of a patch-clamp amplifier (Axopatch-200A; Molecular Devices) for low noise amplification of the photocurrent.

### Data analysis

For the analysis of fluorescence data, baseline fluorescence traces were recorded with no change of voltage during the illumination period. To correct for photobleaching, this baseline trace was low-pass filtered at 1 kHz and subtracted from the fluorescence traces recorded during the application of the voltage protocol. The magnitude of fluorescence signals in traces is expressed as ΔF/F_0%_, where ΔF is the change in the signal amplitude in response to the voltage change and F_0_ is the baseline fluorescence.

Steady-state activation for each individual cell was obtained by normalizing the maximum currents at each voltage with the equation: g = (V − E_Na_)/I_peak_. Available channel fraction after conditioning at different potentials was evaluated at the −20-mV depolarizing pulse. The fluorescence signal amplitude for the fluorescence-voltage (FV) and steady-state inactivation (SSI) fluorescence-voltage (SSI-FV) curves was determined by taking the mean of the signal for at least 5 ms at the greatest displacement of the signal during a 50-ms depolarizing pulse. For SSI-FV, ΔF was calculated as the difference between the signal amplitude measured at the end of the conditioning potential and during −20-mV pulse for at least 5 ms.

Steady-state conductance-voltage (GV), SSI, FV, and SSI-FV curves were obtained by fitting the maximum data points of corresponding traces with a Boltzmann function: y/y_max_ = 1/(1 + exp[(V − V_1/2_)/k)]. VSD deactivation kinetics were assessed by comparing t_10–90%_ from depolarized potentials to baseline potentials. t_10–90_%C were calculated from the time constants of single exponential fits (τ) to the signal by t_10–90_%C = 2.197τ. Statistical comparisons were performed using two sample Student’s *t* tests.

Recovery curves were obtained by fitting the maximum data points of corresponding traces with a sum of exponents function: y = C − A_f_*exp(−t/τ_f_) − A_s_*exp(−t/τ_s_), which accounts for both fast and slower components of recovery.

### Online supplemental material

Fig. S1 shows recovery kinetics of DIII-LFS channel Na^+^ currents were recorded from human Na_V_1.5 channels carrying DIII-LFS mutations. Fig. S2 shows ionic currents and voltage dependence of steady-state parameters of WT and non-LFS mutant channels.

## Results

### VSD kinetics after removal of inactivation with F1486Q

**Figure 1.**
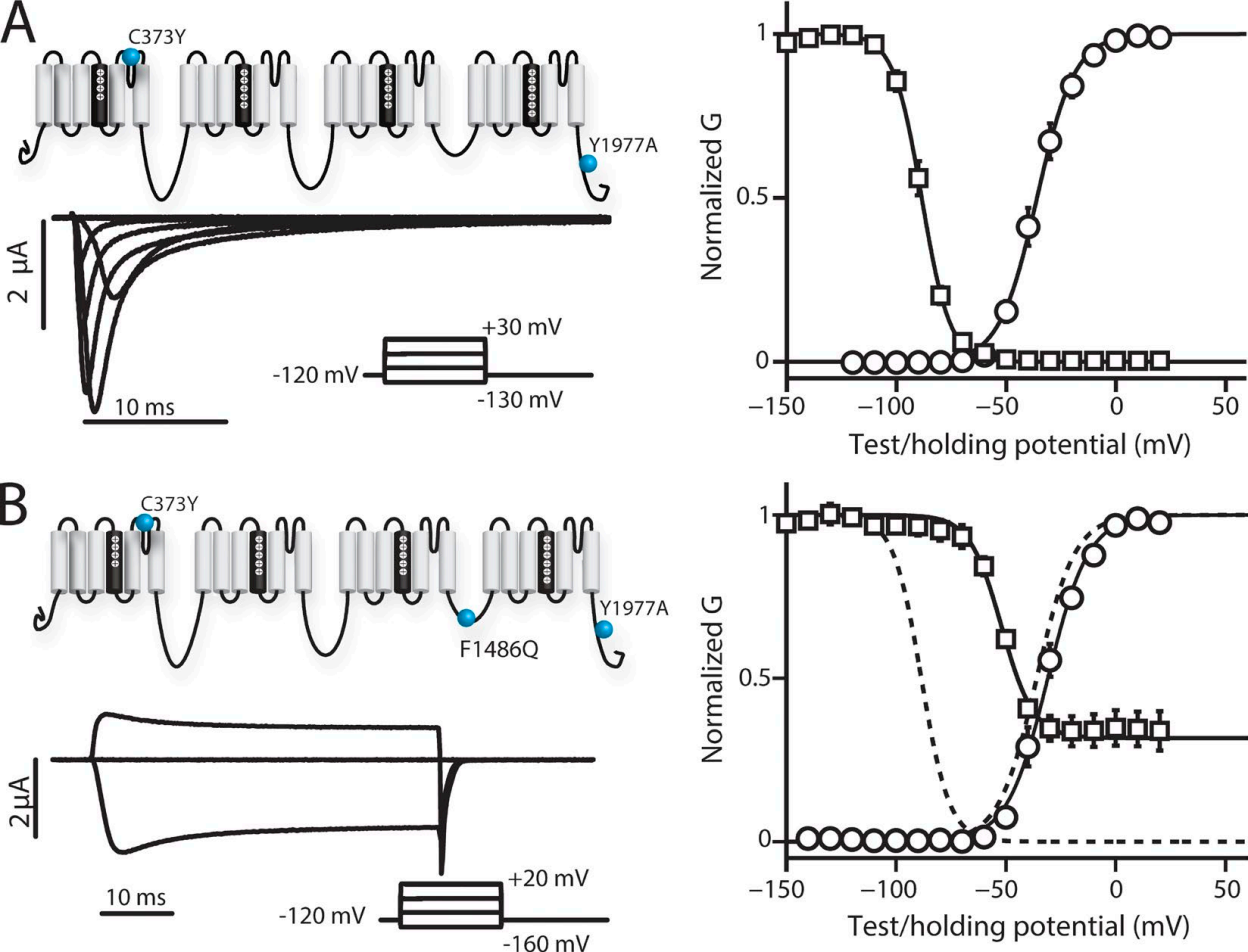
**Ionic currents and voltage dependence of WT-LFS and IQM-LFS channels.** Na^+^ currents were recorded from human Na_V_1.5 channels for WT-LFS and IQM-LFS channels. The mean ± SEM for groups of five to seven cells is reported. To obtain steady-state activation data, cells were held at a potential of −120 mV for 100 ms, depolarized in 20-mV increments for 50 ms where maximum current was measured, and then returned to the original −120-mV holding potential for 50 ms. To obtain the voltage dependence measurements of SSI, cells were held at a potential of −120 mV and depolarized to conditioning potentials in increments of 10 mV for 200 ms. The available channel fraction was assessed by a 20-ms depolarizing pulse to −20 mV. (A, left) Ionic currents from WT channels were recorded during 50-ms-long depolarizing pulses ranging from −150 to 30 mV in 20-mV steps. Current traces corresponding to only −130, −70, −30, and 30 mV are shown for clarity. (right) Voltage dependence curves of steady-state activation (GV, black circles) and SSI (black squares) for WT channels were created. (B, left) Ionic currents from IQM channels were recorded during 50-ms-long depolarizing pulses ranging from −160 to 20 mV in 20-mV steps. Current traces corresponding to only −160, −100, −40, and 20 mV are shown for clarity. (right) Voltage dependence curves of steady-state activation (GV, black circles) and SSI (black squares) for IQM channels were created. Dashed lines represent the corresponding curves for the WT channel for comparison. (A and B) Curves were constructed as described in the Materials and methods section. See Table 1 for Boltzmann fit parameters.

The ability of the IFM motif to participate in hNa_V_1.5 inactivation is strongly impaired by the F1486Q (IQM) mutation (Hartmann et al., 1994). Consistent with these previous results, we observed that introducing IQM into hNa_V_1.5 severely impeded fast inactivation (Fig. 1). In contrast, the voltage dependence of channel conductance (GV curve), measured in response to progressively increasing voltage steps, shows no apparent difference with IQM. We note that inactivation normally truncates the peak of the Na^+^ current, so that even though the voltage dependence of the GV appears similar between WT and IQM, the removal of inactivation alters the measurement. Thus, even though the curves are similar, the G-V relationship is probably altered by removing inactivation. Measuring inactivation showed that after 200-ms pulses to positive potentials, some inactivation was observed by measuring peak current during a test pulse to −20 mV that follows the inactivation-inducing pulses. For the channels that did inactivate, the SSI curve shows a 30-mV rightward shift (Fig. 1 and Table 1).

**Table 1.** Parameters of Boltzmann fits to GV, FV, SSI, and SSI-FV curves

Channel variant	GV/FV					SSI/SSI-FV			
	V_1/2_	k	P_V_	P_k_		V_1/2_	k	P_V_	P_k_
	*mV*	*mV*				*mV*	*mV*		
**GV and SSI**									
WT	−35.3 ± 2.5	8.5 ± 0.5				−83.6 ± 1.6	7.3 ± 0.4		
WT-LFS	−36.0 ± 2.1	8.6 ± 0.4				−88.5 ± 1.3	6.4 ± 0.2		
DI-LFS	−57.7 ± 1.7	7.5 ± 1.4				−78.0 ± 1.4	6.5 ± 0.2		
DII-LFS	−36.4 ± 1.7	8.7 ± 1.1				−82.0 ± 1.5	7.7 ± 1.0		
DIII-LFS	−39.9 ± 2.2	9.5 ± 0.4				−76.2 ± 1.2	6.7 ± 0.2		
DIV-LFS	−33.4 ± 2.5	9.3 ± 0.4				−71.4 ± 1.0	10.0 ± 0.6		
IQM-LFS	−30.9 ± 2.0	9.2 ± 0.2	0.1428	0.2269		−52.0 ± 2.1	7.3 ± 1.9	**3.8 × 10^−6^**	0.1770
A1330P	−36.0 ± 1.9	7.7 ± 0.4	0.8062	0.2825		−74.6 ± 1.1	6.6 ± 0.5	**0.0017**	0.2597
N1325S	−42.5 ± 0.3	2.6 ± 0.7	0.0689	**3.2 × 10^−4^**		−68.3 ± 3.7	5.4 ± 0.2	**0.0045**	**0.0102**
N1659A	−23.7 ± 2.4	9.7 ± 0.3	**0.0085**	0.1639		−52.6 ± 1.5	7.5 ± 1.0	**1.6 × 10^−7^**	0.7790
DI-IQM	−48.7 ± 4.5	11.4 ± 0.5	0.0921	0.0528		−66.2 ± 2.0	3.7 ± 1.0	**0.0023**	**0.0136**
DII-IQM	−32.4 ± 0.8	9.3 ± 0.3	0.0546	0.6054		−49.3 ± 0.8	4.1 ± 0.5	**4.4 × 10^−7^**	0.0547
DIII-IQM	−41.0 ± 2.3	10.8 ± 0.9	0.9502	0.0537		−59.0 ± 1.2	4.0 ± 0.6	**1.5 × 10^−5^**	**1.9 × 10^−4^**
DIV-IQM	−39.3 ± 2.6	11.1 ± 1.0	0.1367	**0.0413**		−58.8 ± 2.6	3.4 ± 0.7	**0.0183**	**4.8 × 10^−4^**
DIII-N1325S	−52.1 ± 1.0	10.2 ± 1.6	**0.0059**	0.2699		−79.9 ± 3.6	7.7 ± 0.7	0.2755	0.1921
DIII-A1330P	−51.1 ± 2.7	9.8 ± 0.6	**0.0081**	0.9331		−79.4 ± 3.9	11.6 ± 0.6	0.3619	**1.4 × 10^−7^**
DIII-N1659A	−37.6 ± 5.9	10.8 ± 0.5	0.5977	0.9349		−42.5 ± 1.4	5.0 ± 0.5	**1.1 × 10^−10^**	**2.3 × 10^−5^**
DIV-N1659A	−4.9 ± 1.1	11.4 ± 0.2	**9.5 × 10^−8^**	**4.6 × 10^−5^**		−48.6 ± 4.0	7.0 ± 0.5	**0.0046**	**9.5 × 10^−4^**
**FV and SSI-FV**									
DI-LFS	−100.4 ± 4.1	18.6 ± 1.0				−83.4 ± 2.6	15.6 ± 1.3		
DII-LFS	−48.6 ± 3.3	20.9 ± 1.6				−48.8 ± 3.5	18.0 ± 0.9		
DIII-LFS	−106.1 ± 3.2	21.7 ± 0.9				−113.6 ± 2.6	15.2 ± 0.6		
DIV-LFS	−61.8 ± 3.6	16.9 ± 1.1				−75.0 ± 2.9	10.0 ± 1.3		
DI-IQM	−91.7 ± 6.1	18.9 ± 3.4	0.2733	0.7766		−91.6 ± 4.2	26.1 ± 2.0	0.1189	**0.0074**
DII-IQM	−50.8 ± 5.0	29.2 ± 4.0	0.6618	0.0833		−57.2 ± 4.0	17.2 ± 3.4	0.2505	0.9400
DIII-IQM	−106.4 ± 5.2	21.7 ± 1.9	0.7518	0.4405		−92.6 ± 2.5	23.1 ± 1.5	**0.0021**	**0.0014**
DIV-IQM	−48.9 ± 3.5	10.8 ± 2.8	**0.0335**	0.1183		−59.2 ± 2.4	5.8 ± 1.2	**0.0032**	**0.0299**
DIII-N1325S	−125.1 ± 1.9	20.7 ± 0.7	**2.6 × 10^−4^**	0.1545		−106.3 ± 2.9	13.9 ± 0.7	0.1328	0.0644
DIII-A1330P	−123.8 ± 3.4	23.6 ± 1.5	**0.0018**	0.8074		−110.9 ± 2.8	15.8 ± 1.3	0.5992	0.9248
DIII-N1659A	−97.0 ± 7.5	24.9 ± 2.8	0.2059	0.6136		−85.0 ± 5.7	23.3 ± 1.5	**0.0019**	**0.0015**
DIV-N1659A	−44.3 ± 2.8	10.6 ± 1.1	**0.0087**	**0.0089**		−48.7 ± 2.3	14.2 ± 2.2	**1.4 × 10^−4^**	0.2343

Midpoints and slope factors from Boltzmann fits y/y_max_ = 1/(1 + exp[(V − V_1/2_)/k)] to steady-state activation and SSI data of ionic currents and fluorescence changes. Mean ± SEM of each parameter for sets of three to seven cells are shown. P-values from two sample *t* tests for mutants compare mutant parameters with corresponding WT parameters. Significant p-values <0.005 are in bold.

Our next aim was to quantify VSD activation with inactivation removed. To accomplish this aim, we introduced IQM into previously developed constructs (large fluorescence signal [LFS]) that have been optimized for VCF by increasing channel expression and reducing background labeling (C373Y and Y1977A). Y1977 is part of a ubiquitination motif that enhances channel recycling and thus reduces channels in the membrane. By removing it, we increase channel expression. C373 is an externally accessible cysteine; thus, the C373Y mutation removes a potential nonspecific site that can be labeled by the fluorescent probe and makes the channel TTX sensitive (Varga et al., 2015). There are four LFS constructs, one for each domain, and each construct contains a cysteine mutation in the extracellular S4 segment of a single domain (DI-LFS V215C, DII-LFS S805C, DIII-LFS M1296C, and DIV-LFS S1618C). To track VSD activation, a fluorophore, TAMRA-MTS, is conjugated to the introduced cysteine (Mannuzzu et al., 1996; Cha et al., 1999). As the VSD changes conformation, the environment around the fluorophore is altered, causing a measurable voltage-dependent change in fluorescence emission (ΔF).

**Figure 2.**
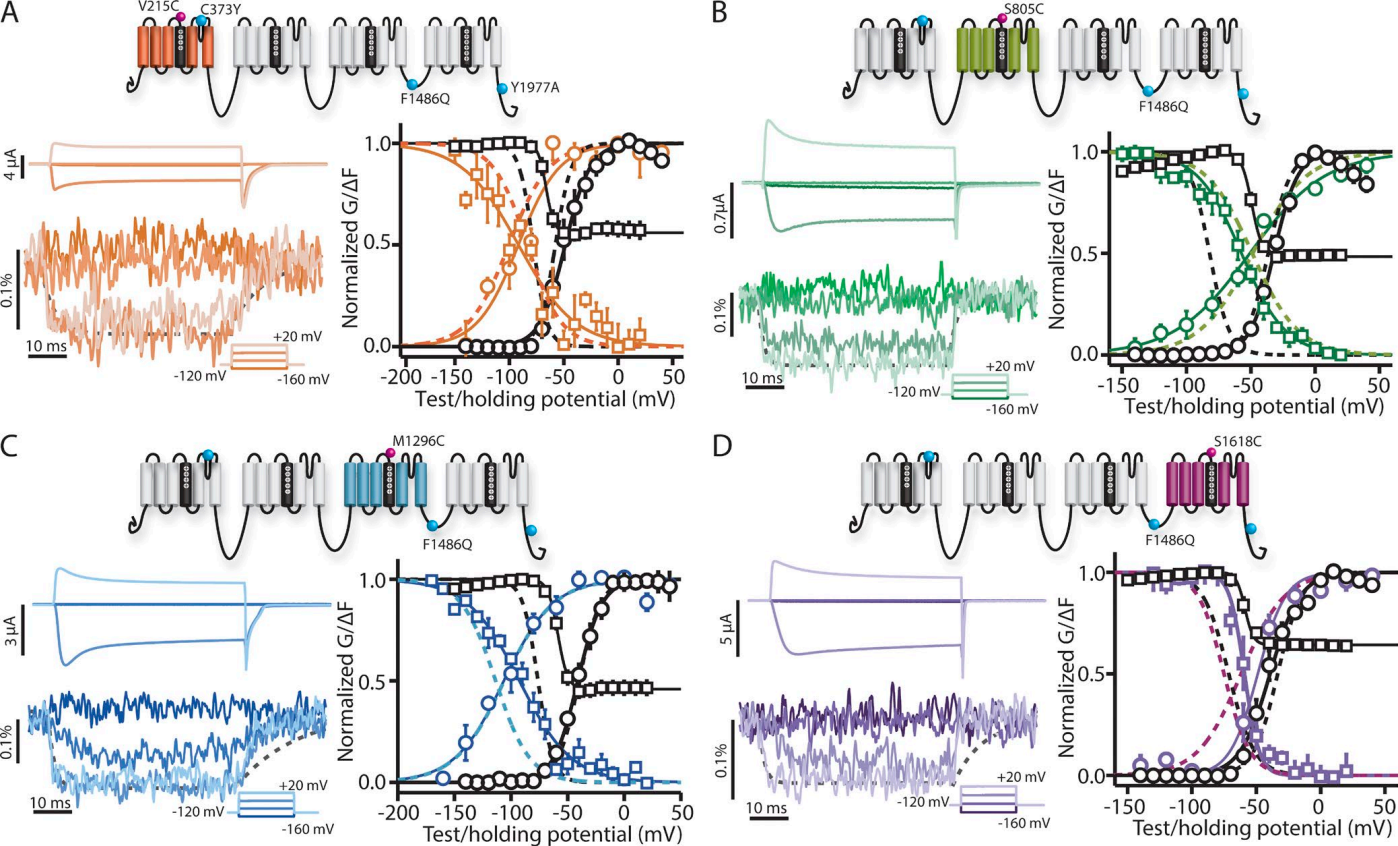
**Steady-state and kinetic properties of ionic currents and fluorescent signals from the DI- to DIV-IQM mutants.** (A–D) Na^+^ currents and fluorescence signals were recorded from human Na_V_1.5 channels carrying DI-IQM (A, orange), DII-IQM (B, green), DIII-IQM (C, blue), or DIV-IQM (D, purple) mutations. GV and SSI curves were obtained as described in Fig. 1. FV curves were measured by the maximum change in ΔF during the depolarizing pulse, and SSI-FV curves were found by measuring the maximum change during the test pulse to −20 mV. Ionic currents (left, top) and fluorescence signals (left, bottom) from DI- to DIV-IQM channels were recorded during 50-ms-long depolarizing pulses ranging from −160 to 80 mV in 20-mV steps. Current and fluorescence traces corresponding to only −160, −100, −40, and 20 mV are shown for clarity. The amounts of current produced and fluorescent signal (in units of ΔF/F) acquired are displayed to the left of their respective traces. Cells with the greatest amount of current produced and fluorescence magnitude are shown. Dashed lines represent fluorescence changes corresponding to DI-, DII-, DIII-, or DIV-LFS channel at 20-mV depolarization (Varga et al., 2015). (right) The mean ± SEM for groups of three to six cells is reported. Voltage dependence curves of steady-state activation (GV, black circles), SSI (black squares), and the corresponding fluorescence signals (colored circles and squares, respectively) for each channel were created. Curves were constructed as described in the Materials and methods section. Dashed lines represent the corresponding curves for the respective DI- to DIV-LFS channel for comparison (Varga et al., 2015). See Table 1 for both LFS and mutant Boltzmann fit parameters.

We have previously shown that the V215 cysteine, used for DI-LFS labeling, shifts the GV curve while minimally affecting gating current (Varga et al., 2015), implying that in spite of the GV shift, the fluorophore still reports on DI-VSD dynamics. Introducing F1486Q into the DI-LFS construct (DI-IQM) impaired inactivation while leaving the GV curve intact, similar to the introduction of IQM in the WT construct (Fig. 2 A and Table 1).

To measure the voltage dependence of DI-VSD activation (FV curve), we recorded fluorescence during the depolarizing pulses of the GV protocol. We also recorded fluorescence during the test pulse of the SSI protocol relative to the fluorescence level at the end of the preceding pulse, SSI-FV curve. The SSI-FV recording was added to test for multiple activated VSD states. For a simple two-state transition (resting to activated), we expect the SSI-FV curve to mirror the FV curve. However, if a VSD transits through multiple open states during the longer (200 ms) SSI protocol, we would expect a differential shift in the SSI-FV curve relative to the FV curve. Comparison of both the DI-IQM FV and SSI-FV curves with those of DI-LFS showed that they were insignificantly affected (Table 1) and that the SSI-FV is a mirror image of the FV curve. Similarly, comparing DI-IQM VSD kinetics with those of DI-LFS (Fig. 2 A, left fluorescence trace, colored and dashed gray lines, respectively) did not show a substantial impact from the introduction of IQM.

Of the four IQM constructs, it was particularly difficult to measure a useful fluorescence signal from DI-IQM. Out of 70 cells recorded, only three survived while expressing enough channels to produce a useful fluorescent signal. This low survivability and expression was evident for all four IQM constructs but was especially low for DI-IQM.

Similar to DI, DII-IQM showed only a modest change in the FV and SSI-FV curves even though SSI was still significantly reduced and shifted (Fig. 2 B and Table 1). These results are consistent with a previous study indicating that the DI- and DII-VSDs are not immobilized by fast inactivation (Cha et al., 1999).

Although the FV curve for DIII-IQM was unchanged in comparison with DIII-LFS, the SSI-FV curve showed a significant shift toward depolarized potentials (Fig. 2 C and Table 1), implying that disruption of inactivation alters the on and off transition rates after a 200-ms hold. As discussed above for DI, the difference in curves is a consequence of the SSI-FV curve being recorded after a 200-ms depolarization and the FV measurement taken after several milliseconds. Thus, this finding implies that the DIII-VSD must be able to enter a different activated state after its initial movement in response to a depolarizing pulse that is affected by the DIII–DIV linker. We also note that in comparison with DIII-LFS, DIII-IQM deactivates more rapidly after 50 ms but still maintains a significant slow component of its deactivation.

DIV-IQM significantly shifts the FV and SSI-FV to depolarized potentials (Fig. 2 D and Table 1). This simultaneous shift, which contrasts with the DIII-VSD, implies that although the IFM motif interacts with the DIV-VSD, DIV-VSD activation can be described with fewer states than DIII-VSD activation. We also observe that with IQM, the DIV-VSD deactivates much more quickly than that of DIV-LFS (Fig. 2 D, left fluorescent trace, colored and dashed gray lines, respectively). The faster deactivation of DIV-IQM implies that interaction of the DIV-VSD with IFM holds the DIV-VSD in the activated conformation.

**Figure 3.**
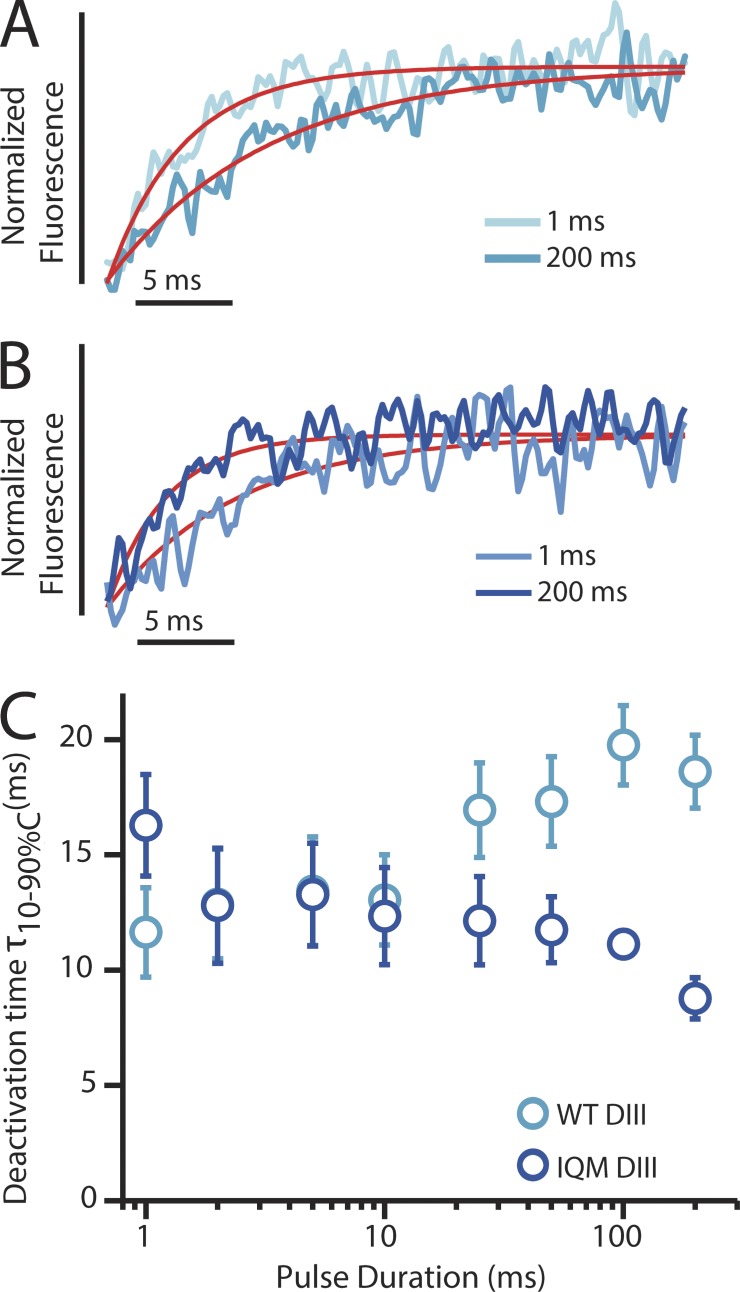
**Kinetic properties of deactivation of the DIII-VSD in both WT and IQM mutant channels.** (A) DIII-LFS fluorescence signals measured after the step back to −120 mV from depolarizing steps to 50 mV for 1 ms (light blue) or 200 ms (dark blue). The exponential fits to the fluorescence traces are shown in red. Deactivation time constants for this cell after 1-ms and 200-ms, −120-mV depolarizations were respectively 3.83 ms and 8.28 ms. (B) DIII-IQM fluorescence signals measured after the step back to −120 mV from depolarizing steps to 50 mV for 1 ms (light blue) or 200 ms (dark blue). The exponential fits to the fluorescence traces are shown in red. Deactivation time constants for this cell after 1-ms and 200-ms, −120-mV depolarizations were respectively 10.91 ms and 5.03 ms. Note the reversal of the 1- and 200-ms rates of recovery when compared with A. (C) 10–90% deactivation time of the fluorescence signal from exponential fits after the step back to −120 mV from depolarizing steps to 50 mV for durations ranging from 1 to 200 ms. DIII-LFS WT times are in light blue, whereas DIII-IQM times are in dark blue. The mean ± SEM for groups of six cells is reported.

Because the DIII-VSD was affected by IQM and its connection to inactivation is not well understood, we probed its kinetics in more detail. Deactivation of the DIII-VSD in WT channels slows after long depolarizations (Fig. 3 and Table 2; Varga et al., 2015). We hypothesized that the IFM motif modulates this slowing, as it has been suggested to interact with the DIII S4–S5 linker in Na_V_1.2 (Smith and Goldin, 1997). In stark contrast to DIII-WT, DIII-IQM notably speeds deactivation after long depolarizations (Fig. 3 B), consistent with the depolarizing shift observed in the DIII SSI-FV curve shown in Fig. 2. However, the DIII-VSD deactivation rate in the 10-ms domain, where fast inactivation occurs, is not affected (Fig. 3 C). This finding suggests that the DIII-VSD does not interact with the DIII–DIV linker in the fast inactivation time domain, but instead interacts after pulses that last hundreds of milliseconds. This time domain is of particular importance to human cardiac physiology because ventricular and atrial action potentials have durations of 100–400 ms.

**Table 2.** Parameters of sum of exponential fits for recovery curves

Channel variant	C	A_f_	τ_f_	A_s_	τ_s_
			*ms*		*ms*
**200-ms pulse**					
DIII-LFS	0.967 ± 0.006	0.978 ± 0.049	4.443 ± 0.615	0.183 ± 0.019	83.5 ± 17.1
DIII-N1325S	0.990 ± 0.005	1.042 ± 0.065	3.237 ± 0.550	0.172 ± 0.026	22.9 ± 6.3
DIII-A1330P	0.989 ± 0.004	1.006 ± 0.019	3.952 ± 0.254	0.141 ± 0.034	44.0 ± 5.6
DIII-N1659A	0.995 ± 0.005	0.528 ± 0.165	1.906 ± 0.438	0.115 ± 0.010	44.3 ± 15.0
DIII-IQM	0.998 ± 0.001	0.532 ± 0.063	1.472 ± 0.144	0.069 ± 0.011	24.5 ± 6.3
**10-ms pulse**					
DIII-LFS	0.991 ± 0.003	1.000 ± 0.034	5.346 ± 0.455	0.087 ± 0.008	64.6 ± 10.5
DIII-N1325S	0.993 ± 0.007	0.983 ± 0.019	5.073 ± 0.406	0.114 ± 0.008	52.0 ± 10.1
DIII-A1330P	0.993 ± 0.001	1.121 ± 0.077	2.322 ± 0.186	0.114 ± 0.007	15.6 ± 1.6
DIII-N1659A	0.989 ± 0.011	0.493 ± 0.153	0.900 ± 0.485	0.035 ± 0.020	32.1 ± 17.9

As described in the Materials and methods section, time dependence of recovery saw fit using a sum of exponentials with the following equation: fraction recovered y = C − A_f_*exp(−t/τ_f_) − A_s_*exp(−t/τ_s_). Recovery after 10- and 200-ms depolarizing pulses are both shown here. Mean ± SEM of each parameter for sets of three to seven cells are shown.

### Assessing the role of the DIII and DIV S4–S5 linkers

**Figure 4.**
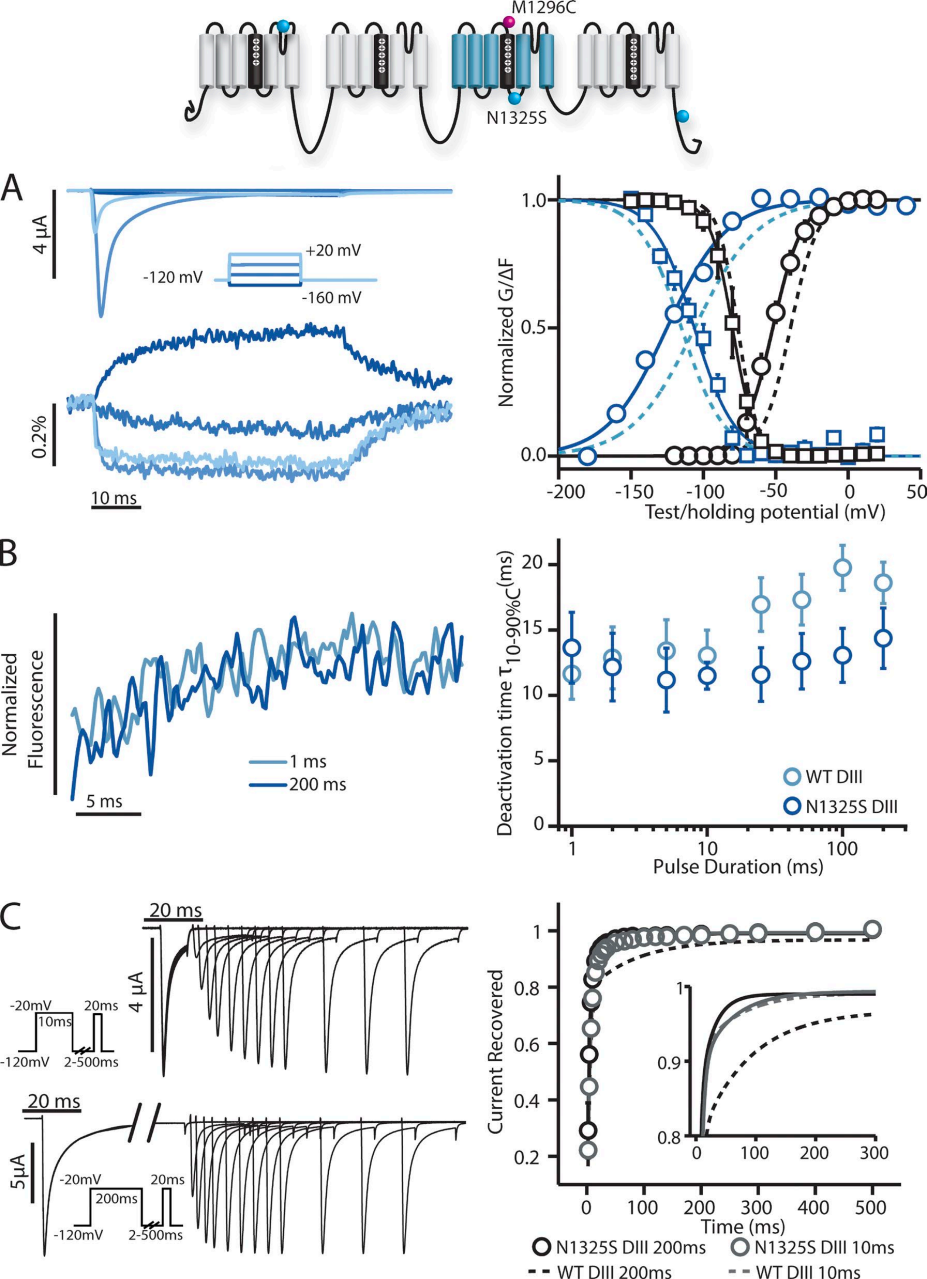
**Steady-state and kinetic properties of ionic currents and fluorescent signals from the DIII-N1325S long QT syndrome type 3 mutant.** Na^+^ currents and fluorescence signals were recorded from human Na_V_1.5 channels carrying DIII-N1325S mutations. The mean ± SEM for groups of three to six cells is reported. Activation, inactivation, and fluorescence curves were obtained via the protocols described in Figs. 1 and 2. To obtain recovery data, cells were held at a potential of −120 mV for 50 ms and depolarized to −20 mV for either 10 or 200 ms. The potential was then returned to −120 mV for varying recovery durations of 2–500 ms before being depolarized to −20 mV again for 20 ms. The fraction of current recovered was measured as the peak current during this −20-mV hold divided by the peak current of the first −20-mV hold. The potential was finally returned to the original −120-mV holding potential for 50 ms. (A, left) Ionic currents from DIII-N1325S channels were recorded during 50-ms-long depolarizing pulses ranging from −160 to 20 mV in 20-mV steps. Current traces corresponding to only −160, −100, −40, and 20 mV are shown for clarity. (right) Voltage dependence curves of steady-state activation (GV, black circles), SSI (black squares), and the corresponding fluorescence signals (colored circles and squares, respectively) for DIII-N1325S channels were created. See Table 1 for Boltzmann fit parameters. (B, left) DIII-N1325S fluorescence signals measured after the step back to −120 mV from depolarizing steps to 50 mV for 1 (light blue) or 200 ms (dark blue). (right) 10–90% deactivation time of the fluorescence signal from exponential fits after the step back to −120 mV from depolarizing steps to 50 mV for durations ranging from 1 to 200 ms. DIII-LFS WT times are in light blue, whereas DIII-N1325S times are in dark blue. (C, left) Ionic currents from DIII-N1325S channels were recorded with the protocol used for the IQM mutations. The initial −20-mV depolarizing pulse was held for either 10 (top) or 200 ms (bottom). (right) Time dependence of fraction of current recovered for DIII-N1325S after a 10-ms depolarizing pulse (gray) or 200-ms depolarizing pulse (black). The smaller subplot only shows the fitted curves for time dependence of recovery, with solid lines representing DIII-N1325S recovery. See Table 2 for exponential parameters. (A and C) Curves were constructed as described in the Materials and methods section. Dashed lines represent the corresponding curves for the DIII-LFS channel for comparison.

Next, we focused on the DIII S4–S5 linker by assessing how the LQT3 DIII S4–S5 linker mutation N1325S affects DIII-VSD activation. Previously, N1325S has been shown to increase late Na^+^ current by facilitating late openings (Dumaine et al., 1996), a pathology that is also observed in transgenic N1325S mice (Tian et al., 2004). We probed DIII-VSD kinetics in the presence of the N1325S mutation (DIII-N1325S) with the same protocols used to characterize the IQM mutation. Consistent with a previous study (Dumaine et al., 1996), N1325S produced a large hyperpolarizing GV curve shift and a minimal shift in SSI (Table 1 and Fig. 4 A, right graph, black lines). N1325S also hyperpolarizes the DIII-VSD FV curve but does not significantly shift the SSI-FV curve (Table 1 and Fig. 4 A, right graph, colored lines). As discussed above, the shifting of only one of the two fluorescence curves indicates that the DIII-VSD must traverse multiple states as it activates. The result of N1325S shifting only the FV curve supports this hypothesis and implies that N1325S and IQM, both of which affect DIII, affect different DIII-VSD activation transitions.

We further probed the connection between the DIII-VSD and inactivation by measuring recovery from inactivation after 10- and 200-ms pulses and observed a striking difference between N1325S and WT, where a slower component of inactivation recovery had been eliminated by the mutations. For WT channels, the recovery from inactivation after a 200-ms depolarizing pulse is significantly slowed compared with the recovery rate after a 10-ms pulse, suggesting during the longer pulse, channels enter a deeper inactivation state from which it takes longer to recover. In contrast, for N1325S channels, the recovery rate after 200 ms remains the same as after the 10-ms depolarizing pulse, showing that N1325S eliminated the deeper inactivated state (Fig. 4 C). Interestingly, there is also no difference between the deactivation rate of the DIII-VSD after 10 or 200 ms (Fig. 4 B).

**Figure 5.**
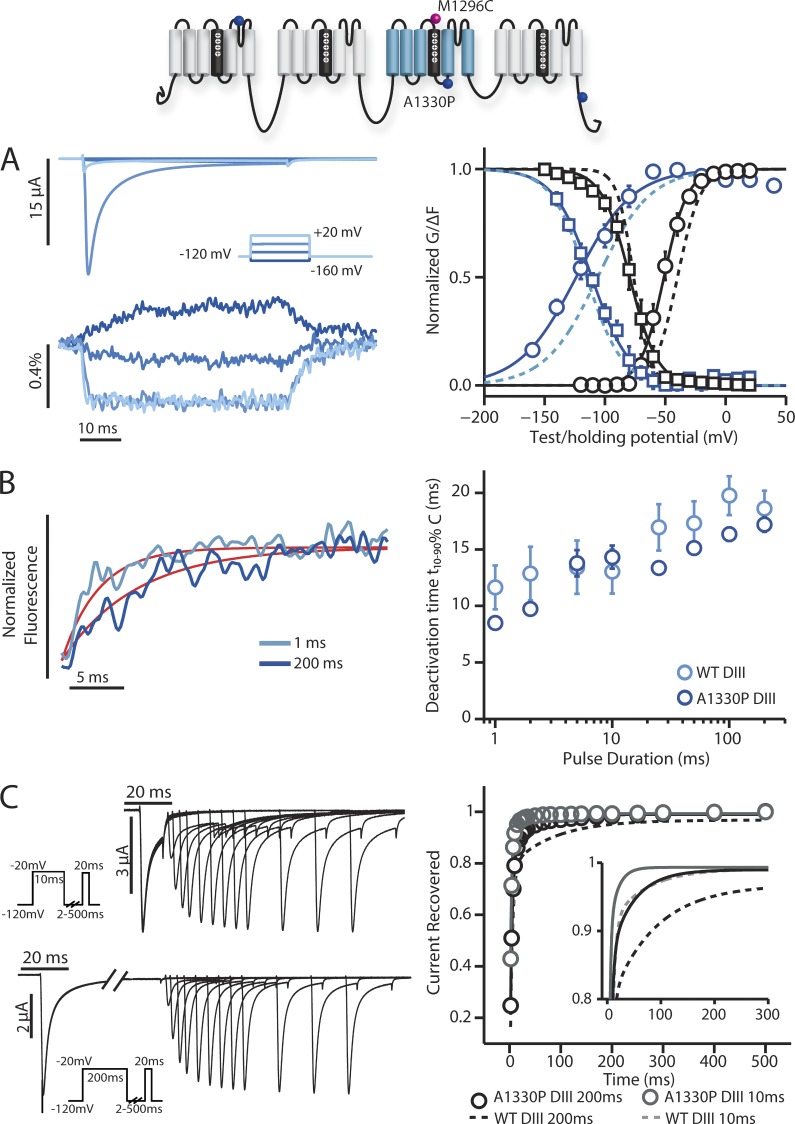
**Steady-state and kinetic properties of ionic currents and fluorescent signals from the DIII-A1330P long QT syndrome type 3 mutant.** Na^+^ currents and fluorescence signals were recorded from human Na_V_1.5 channels carrying DIII-A1330P mutations. The mean ± SEM for groups of three to six cells is reported. Activation, inactivation, and fluorescence curves were obtained via the protocols described in Figs. 1 and 2. Recovery curves were obtained via the protocols described in Fig. 4. (A, left) Ionic currents from DIII-A1330P channels were recorded during 50-ms-long depolarizing pulses ranging from −160 to 20 mV in 20-mV steps. Current traces corresponding to only −160, −100, −40, and 20 mV are shown for clarity. (right) Voltage dependence curves of steady-state activation (GV, black circles), SSI (black squares), and the corresponding fluorescence signals (colored circles and squares, respectively) for DIII-A1330P channels were created. See Table 1 for Boltzmann fit parameters. (B, left) DIII-A1330P fluorescence signals measured after the step back to −120 mV from depolarizing steps to 50 mV for 1 (light blue) or 200 ms (dark blue). The exponential fits to the fluorescence traces are shown in red. Deactivation time constants for this cell after 1-ms and 200-ms, −120-mV depolarizations were respectively 3.37 ms and 6.82 ms. (right) 10–90% deactivation time of the fluorescence signal from exponential fits after the step back to −120 mV from depolarizing steps to 50 mV for durations ranging from 1 to 200 ms. DIII-LFS WT times are in light blue, whereas DIII-A1330P times are in dark blue. (C, left) Ionic currents from DIII-A1330P channels were recorded with the protocol described in Fig. 4. The initial −20-mV depolarizing pulse was held for either 10 (top) or 200 ms (bottom). (right) Time dependence of fraction of current recovered for DIII-A1330P after a 10-ms depolarizing pulse (gray) or 200-ms depolarizing pulse (black). The smaller subplot only shows the fitted curves for time dependence of recovery, with solid lines representing DIII-A1330P recovery. See Table 2 for exponential parameters. (A and C) Curves were constructed as described in the Materials and methods section. Dashed lines represent the corresponding curves for the DIII-LFS channel for comparison.

We next investigated the A1330P mutation, which has been reported to shift the V_1/2_ SSI by 8 mV when expressed in HEK 293T cells (Wedekind et al., 2001). Contrary to our expectation, we did not observe a substantial shift in SSI, but instead observed an ionic current phenotype that was similar to N1325S, where activation was moderately shifted to negative potentials (Fig. 5 A and Table 1). Similarly, DIII-VSD activation was also left-shifted. However, in contrast to N1325S, which showed no difference between the rate of DIII-VSD deactivation at 1 and 200 ms, in A1330P this dependence was mostly preserved (Fig. 5 B). Measurement of recovery from inactivation showed that recovery kinetics were affected but not to the same extent as N1325S (Fig. 5 C).

Because IQM also affected the voltage dependence of DIV-VSD activation and inactivation, we hypothesized that perturbing DIV-VSD interaction with inactivation would affect its kinetics. A previous study in rNa_V_1.2 suggests that the N1659 position on the DIV S4–S5 linker potentially interacts with the IFM motif on the DIII–DIV linker (McPhee et al., 1998). To probe DIII-VSD and DIV-VSD interaction and test our hypothesis, we incorporated the N1659A mutation into both the DIV-LFS and DIII-LFS constructs.

**Figure 6.**
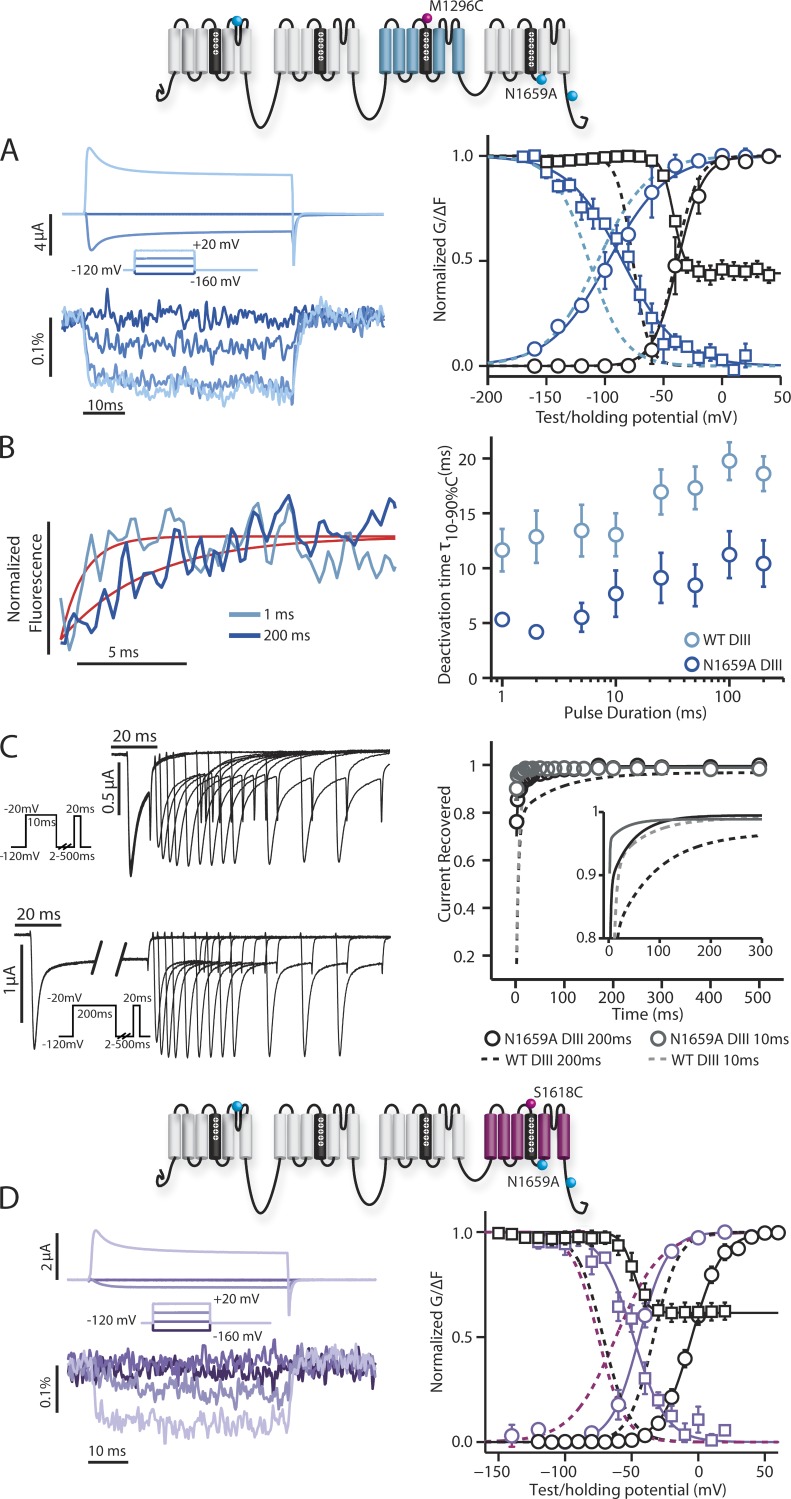
**Steady-state and kinetic properties of ionic currents and fluorescent signals from the DIII-N1659A and DIV-N1659A mutant.** Na^+^ currents and fluorescence signals were recorded from human Na_V_1.5 channels carrying DIII-N1659A or DIV-N1659A mutations. The mean ± SEM for groups of three to six cells is reported. Activation, inactivation, and fluorescence curves were obtained via the protocols described in Figs. 1 and 2. Recovery curves were obtained via the protocols described in Fig. 4. (A, left) Ionic currents from DIII-N1659A channels were recorded during 50-ms-long depolarizing pulses ranging from −160 to 20 mV in 20-mV steps. Current traces corresponding to only −160, −100, −40, and 20 mV are shown for clarity. (right) Voltage dependence curves of steady-state activation (GV, black circles), SSI (black squares), and the corresponding fluorescence signals (colored circles and squares, respectively) for DIII-N1659A channels were created. (B, left) DIII-N1659A fluorescence signals measured after the step back to −120 mV from depolarizing steps to 50 mV for 1 (light blue) or 200 ms (dark blue). The exponential fits to the fluorescence traces are shown in red. Deactivation time constants for this cell after 1-ms and 200-ms, −120-mV depolarizations were respectively 1.04 ms and 3.97 ms. (right) 10–90% deactivation time of the fluorescence signal from exponential fits after the step back to −120 mV from depolarizing steps to 50 mV for durations ranging from 1 to 200 ms. DIII-LFS WT times are in light blue, whereas DIII-N1659A times are in dark blue. (C, left) Ionic currents from DIII-N1659A channels were recorded with the protocol described in Fig. 4. The initial −20-mV depolarizing pulse was held for either 10 (top) or 200 ms (bottom). (right) Time dependence of fraction of current recovered for DIII-N1659A after a 10-ms depolarizing pulse (gray) or 200-ms depolarizing pulse (black). The smaller subplot only shows the fitted curves for time dependence of recovery, with solid lines representing DIII-N1659A recovery. See Table 2 for exponential parameters. (A and C) Curves were constructed as described in the Materials and methods section. Dashed lines represent the corresponding curves for the DIII-LFS channel for comparison. (D, left) Ionic currents from DIV-N1659A channels were recorded during 50-ms-long depolarizing pulses ranging from −160 to 20 mV in 20-mV steps. Current traces corresponding to only −160, −100, −40, and 20 mV are shown for clarity. (right) Voltage dependence curves of steady-state activation (GV, black circles), SSI (black squares), and the corresponding fluorescence signals (colored circles and squares, respectively) for DIV-N1659A channels were created. Curves were constructed as described in the Materials and methods section. Dashed lines represent the corresponding curves for the DIV-LFS channel for comparison. (A and D) See Table 1 for Boltzmann fit parameters.

With this mutation, we observed a very significant disruption of inactivation. DIII-N1659A exhibits a modest FV and a large depolarizing shift in SSI-FV curve. More striking is the rapidity of the deactivation of the DIII-VSD (Fig. 6 A), which more strongly resembles the deactivation of the DII-VSD (Fig. 2 B, left fluorescent trace) than the WT DIII-VSD (Fig. 2 C, left fluorescent trace, gray dashed line). This result suggests that the slow deactivation of the DIII-VSD is largely dependent on the conformation of DIV. Like DIII- and in contrast to DIII-IQM, DIII-N1659A also exhibits slower deactivation after long pulses, suggesting that its DIII-VSD can still interact with the DIII–DIV linker (Fig. 6 B).

The inactivation phenotype of DIV-N1659A also shows strong impairment, consistent with a right-shifted DIV-FV curve and rapid deactivation of the DIV-VSD (Fig. 6 D), akin to DIV-IQM. This similarity between the DIV-IQM and the DIV-N1659A phenotypes supports a direct interaction of the DIV-VSD with the DIII–DIV linker (McPhee et al., 1998). We note that this construct shows a very significant right shift in the GV curve that was substantially larger than the N1659A mutation in the WT background (Fig. S2), which prevents us from making significant conclusions about how the mutation affects channel activation.

**Figure 7.**
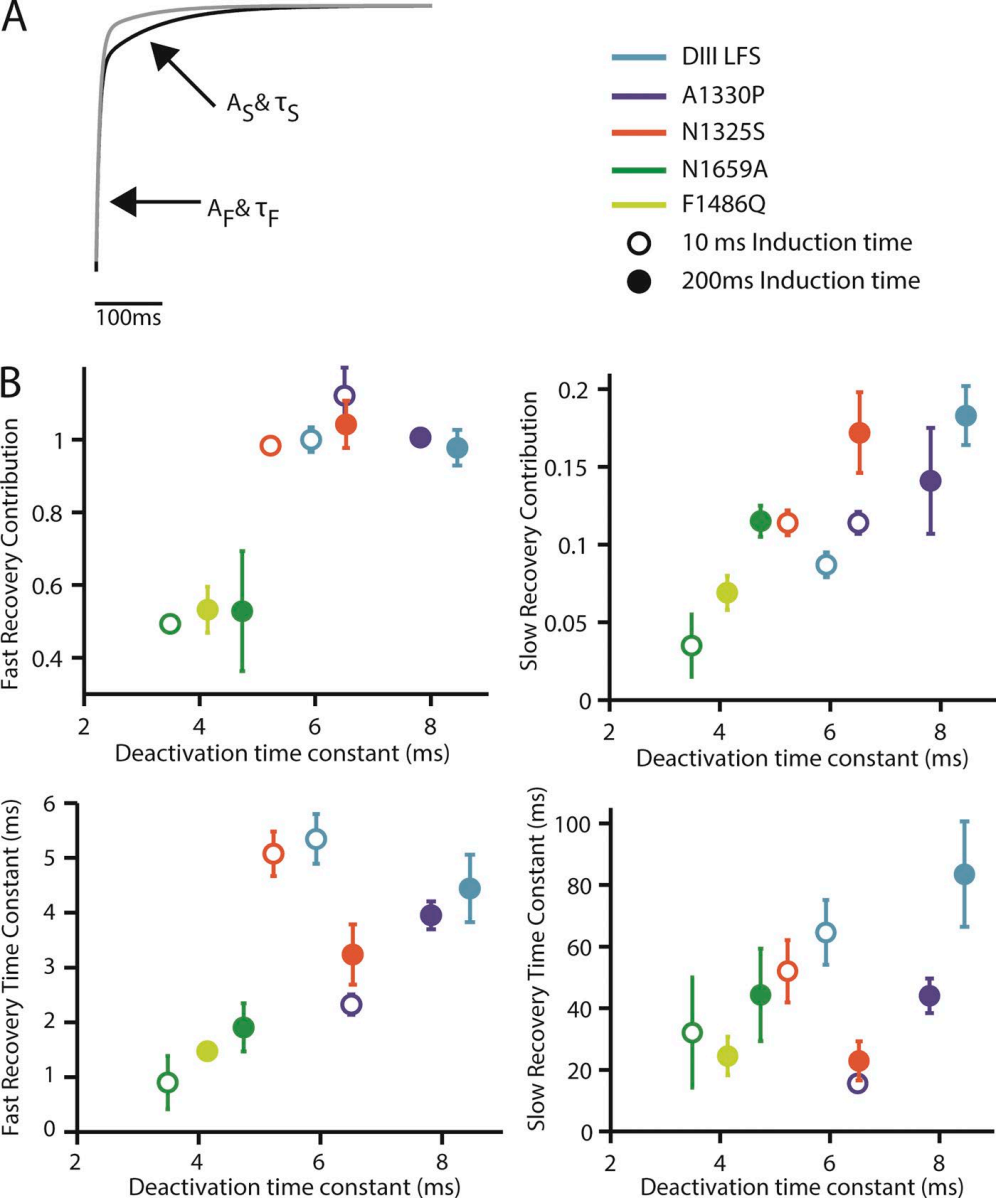
**Comparison of DIII-VSD deactivation kinetics with recovery kinetics.** As described in the Materials and methods section, time dependence of recovery saw fit using a sum of exponentials with the following equation: fraction recovered y = C − A_f_*exp(−t/τ_f_) − A_s_*exp(−t/τ_s_). (A) Representative recovery from WT, displaying each component. A_f_ and τ_f_ more heavily contribute to recovery after shorter recovery durations, whereas A_s_ and τ_s_ more heavily contribute to recovery after longer recovery durations. (B) All four of the above parameters for each mutant were plotted against DIII-VSD deactivation time after 10-ms (open circles) and 200-ms (closed circles) pulses. The legend describes the color that each mutant’s deactivation or recovery parameters correspond to on each graph.

While perturbing the voltage-dependent kinetics of the DIII-VSD with the IQM, N1325S, A1330P, and N1659A mutations, we have noted a correlation between recovery from inactivation and the deactivation rate of the DIII-VSD. In Fig. 7, we plot time constants of recovery from inactivation fit with two exponentials against the deactivation rate of the DIII-VSD (recovery data for DIII-LFS is in Fig. S1). For the fast component of recovery, we observe a strong correlation between DIII-VSD deactivation rate and the rate of recovery for 200-ms pulses but not for 10-ms pulses. This strong correlation suggests that the DIII-VSD plays a dominant role in determining inactivation recovery after longer, action potential–length, pulses. We also observed a correlation between the magnitude of the slower, intermediate component of recovery and DIII-VSD deactivation rate, suggesting allosteric regulation where the activated state of the DIII-VSD promotes entry into the intermediate inactivated state.

## Discussion

We have shown that disruption of inactivation with the F1486Q and N1659A mutations greatly speeds the rate of DIV-VSD recovery (Figs. 2 and 6), supporting a direct interaction between the intracellular DIV S4–S5 and DIII–DIV linkers that determines the onset of inactivation (McPhee et al., 1998; Capes et al., 2013). In contrast, IQM causes the DIII-VSD to deactivate much more rapidly (Fig. 3) only after prolonged depolarization (∼100 ms). After these longer pulses, a very strong correlation is observed between the DIII-VSD deactivation rate and the fast rate of recovery from inactivation (Fig. 7). A second correlation is also observed between the DIII-VSD deactivation rate and the magnitude of the slow recovery component as exemplified by the N1325S mutation, further highlighting the role of the DIII-VSD in regulating inactivation.

**Figure 8.**
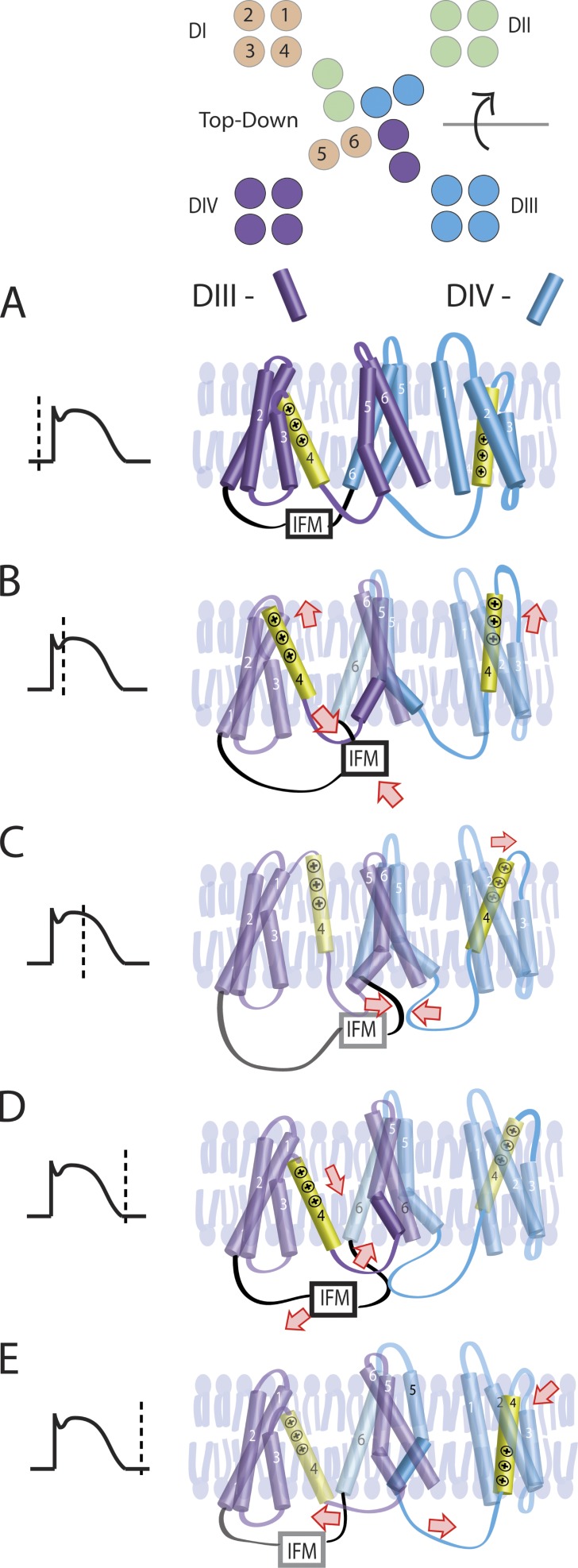
**A working model for DIII-VSD and DIV-VSD regulation of inactivation.** (A) At rest, the DIII- and DIV-VSDs are in the resting conformation, the DIII–DIV linker is unbound, and the channel is not inactivated. (B) Upon depolarization, the DIII- and DIV-VSDs activate within 10 ms (Fig. 2), facilitating interaction between the DIII–DIV linker and the DIV-VSD. (C) After ∼100 ms, the DIII–DIV linker undergoes a second, IFM-dependent interaction with the DIII-VSD that stabilizes its activated conformation. (D) Once the cell returns to its resting potential, the DIII–DIV linker first dissociates from the DIV-VSD. (E) Later, the DIII–DIV linker dissociates from the DIII-VSD, allowing recovery from inactivation to occur.

A potential model that accounts for these results is illustrated in Fig. 8. In this model, activation of the DIV-VSD mediates inactivation onset via a direct interaction with the IFM motif and the DIV S4–S5 linker, consistent with F1486Q (IQM) and N1659A results (Figs. 2 and 6). Over ∼100 ms, an IFM-dependent interaction of the DIII–DIV linker and the DIII-VSD occurs that stabilizes the activated DIII-VSD conformation, as indicated by the time and IFM dependence of the DIII-VSD deactivation rate (Fig. 3). For longer pulses, this secondary interaction with the DIII-VSD is the primary determinant of the inactivation recovery rate, as implied by the strong correlation between DIII-VSD deactivation and inactivation recovery after 200-ms pulses (Fig. 7). During a cardiac action potential, the DIV-VSD will determine inactivation onset, whereas the DIII-VSD will regulate its recovery. Thus, this model implies that the DIII-VSD will determine Na^+^ channel availability immediately after an action potential to mediate refractoriness of excitability, a critical determinant of arrhythmia susceptibility (Mines, 1913).

### Gating charges and inactivation

Early gating current results by Armstrong and Bezanilla (1977) showed that a significant fraction of the gating charge is immobilized with a time and voltage dependence that correlates with that of inactivation. Now that it is well known that the charged S4 segments within the Na_V_ channel VSDs carry most of this charge, it is clear that this was the first evidence of direct VSD interaction with inactivation. Subsequent work, applying VCF to observe the Na_V_1.4 VSDs, has shown that DIII- and DIV-VSD immobilization is correlated with the onset of inactivation (Cha et al., 1999). More recently, neutralization of critical S4 arginines within each VSD revealed a tight connection between DIV-VSD activation and channel inactivation (Capes et al., 2013).

Our results with IQM are consistent with these previous findings, revealing that the voltage dependence of DIV-VSD activation is shifted to depolarized potentials by IQM disruption of inactivation and that its deactivation is much more rapid (Fig. 2 D). This shift and rapid deactivation imply IFM-mediated stabilization of the DIV-VSD–activated state in WT channels. Thus, when this stabilization is removed by IQM, higher potentials are required to activate the DIV-VSD, and its return to the resting conformation is facilitated.

In contrast to DIV, DIII-VSD interaction with IQM is more complex and shows no shift after short pulses and a significant rightward shift in the SSI-FV curve after the longer 200-ms pulses that are used to monitor inactivation (Fig. 2 C). This type of behavior suggests that the DIII-VSD enters a deep state after prolonged depolarization. Consistent with multiple DIII-VSD states, we have previously observed that the time constant of DIII-VSD deactivation depends on pulse duration (Varga et al., 2015), with longer pulses slowing the return of the DIII-VSD to its resting state. IQM severely disrupted this dependence, causing the DIII-VSD to recover more rapidly after prolonged depolarization (Fig. 3) and implying an interaction with the DIII–DIV linker in the 100-ms time domain.

The N1659A mutation significantly enhanced the rate of DIII-VSD return to the resting state, in contrast to IQM where immobilization was not removed until the application of long pulses. This difference between N1659A and IQM implies that after short pulses, DIII-VSD immobilization is primarily determined by the activation of the DIV-VSD rather than by DIII–DIV linker–mediated inactivation.

### Physiological significance

After excitation propagates through the atria and ventricles, it normally terminates by colliding with itself at the apex of the heart. This inability of the wave front to continue to propagate past recently excited tissue is largely caused by inactivation of Na_V_1.5 channels, a condition that persists after excitation, known as the refractory period. When Na_V_1.5 channels have recovered from inactivation, the myocardium is no longer refractory and can be re-excited by subsequent excitatory waves.

The ability of the myocardium to sustain a reentrant arrhythmia depends on three parameters: (1) the length of the pathway that the reentrant excitation can travel, (2) the conduction velocity of excitation, and (3) the refractory period after excitation. If the pathway is too short, conduction velocity is too fast or refractoriness too prolonged, then the arrhythmia cannot be sustained (Mines, 1913). Our results show that after action potential length pulses, the deactivation rate of the DIII-VSD strongly correlates to the recovery kinetics of Na_V_1.5 inactivation (Fig. 7). This result implies that the DIII-VSD regulates not only recovery from inactivation but also the duration of the refractory period and the ability of the heart to sustain a reentrant wave. Intriguingly, local anesthetics, whose derivatives are often used to treat arrhythmia patients also interact with the DIII-VSD (Sheets and Hanck, 2003; Arcisio-Miranda et al., 2010), suggesting a dynamic interaction between inactivation recovery and anti-arrhythmic drug blockade that is highly likely to impact the therapeutic efficacy of these molecules.

### Molecular pathology of inherited mutations

Hundreds of inherited mutations to *SCN5A* that predispose patients to arrhythmia have been discovered, and these mutations are often pathological because of a gain or loss of inactivation. The positions of these mutations can suggest channel locales that are connected to inactivation. For example, three Brugada syndrome (channel loss-of-function) and seven long QT syndrome type 3 (channel gain-of-function) mutations can be found on the 19–amino acid DIII S4–S5 linker (UniProt Consortium, 2015). We probed the role of two of these mutations in disrupting inactivation and DIII-VSD activation by introducing two long QT–causing mutations, N1325S and A1330P. In contrast to IQM, the N1325S mutation shifted the activation of the DIII-VSD toward negative potentials during the GV protocol, which correlated with a shift in the GV curve (Fig. 4), consistent with previous work in rNa_V_1.4 showing a connection between the DIII-VSD and channel activation (Muroi et al., 2010). The primary change to inactivation was evident in the recovery kinetics.

As discussed in the section on physiological significance, Na_V_1.5 inactivation recovery kinetics play a key role in determining the refractory period and the ability of the heart to sustain arrhythmia. As can be seen most clearly in Fig. 7, both the N1325S and A1330P mutations speed inactivation recovery. This finding implies that the increased rate of DIII-VSD recovery will also hasten Na_V_1.5 inactivation recovery and reduce the refractory period. Coupled to increased late current as in N1325S, the more rapid recovery will produce a highly pro-arrhythmic substrate that is more likely to trigger and sustain an arrhythmia.

### Limitations

For the N1659A DIV-VSD mutant, we observed a very large depolarized shift in the GV curve that was not seen when probing the DIII-VSD or the mutation in a WT background. Thus, we expect that this shift is a consequence of inserting a cysteine mutation and TAMRA-MTS fluorophore within the DIV-VSD. Similarly, the DIII-LFS construct also showed a different A1330P and N1325S inactivation phenotype than the WT (Fig. S2). Together, these results suggest that subtle kinetic changes that are endowed by mutations on the VSD that is being probed will not be well resolved with VCF.

### Conclusions

Together, our results are consistent with dual regulation of inactivation by the DIV-VSD that modulates onset and the DIII-VSD that determines recovery. By regulating inactivation recovery, the DIII-VSD will also significantly determine the refractory period after myocardial excitation, an important parameter that impacts arrhythmia susceptibility. Mutations that alter this DIII-VSD regulation of inactivation recovery are likely to predispose patients to arrhythmia.

## Supplementary Material

Supplemental Materials (PDF)
