# Supplementary material for: Regulation of Na+ channel inactivation by the DIII and DIV voltage-sensing domains
Source: J Gen Physiol. 2017 Mar 6;149(3):389–403. doi: 10.1085/jgp.201611678 (PMC5339511; doi:10.1085/jgp.201611678)
Supplement: Supplemental Materials (PDF) [file JGP_201611678_sm.pdf]

The Journal of General Physiology

SUPPLEMENTAL MATERIAL

Hsu et al., https://doi.org/10.1085/jgp.201611678

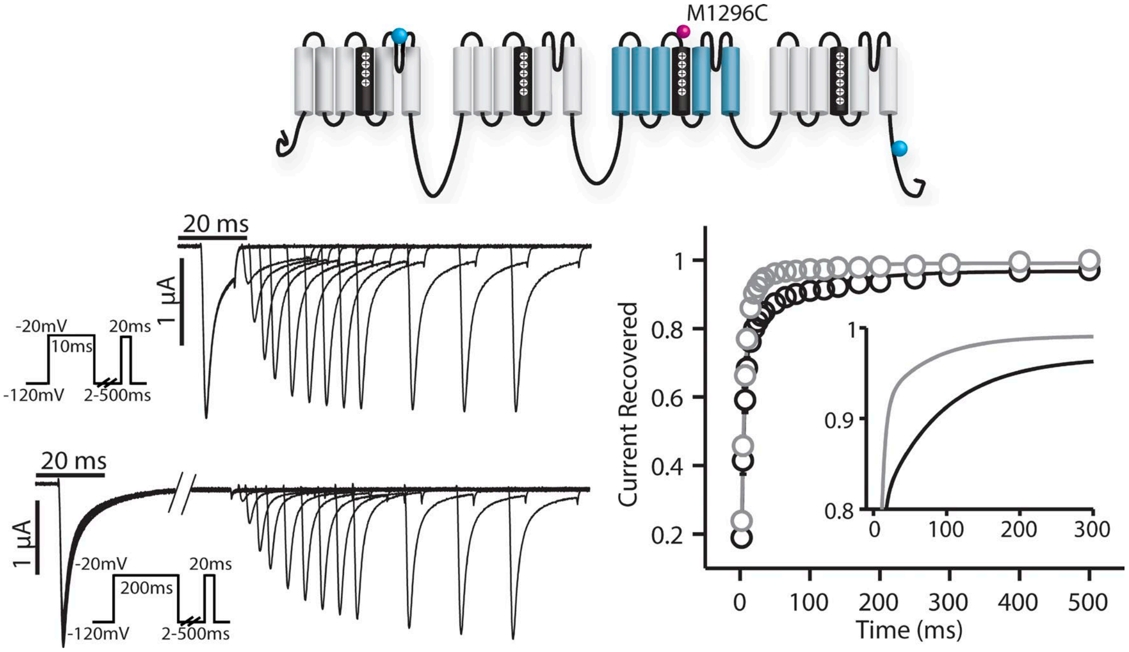

Figure S1. **Recovery kinetics of DIII-LFS channel Na^+ currents were recorded from human $Na_V1.5$ channels carrying DIII-LFS mutations.** The mean ± SEM for seven cells is reported. Recovery curves were obtained via the protocols described in Fig. 4. (left) Ionic currents from DIII-N1659A channels were recorded with the protocol described in Fig. 4. The initial −20-mV depolarizing pulse was held for either 10 (top) or 200 ms (bottom). (right) Time dependence of fraction of current recovered for DIII-LFS after a 10-ms depolarizing pulse (gray) or 200-ms depolarizing pulse (black). The smaller subplot only shows the fitted curves for time dependence of recovery. Curves were constructed as described in the Materials and methods section. See Table 2 for exponential parameters.

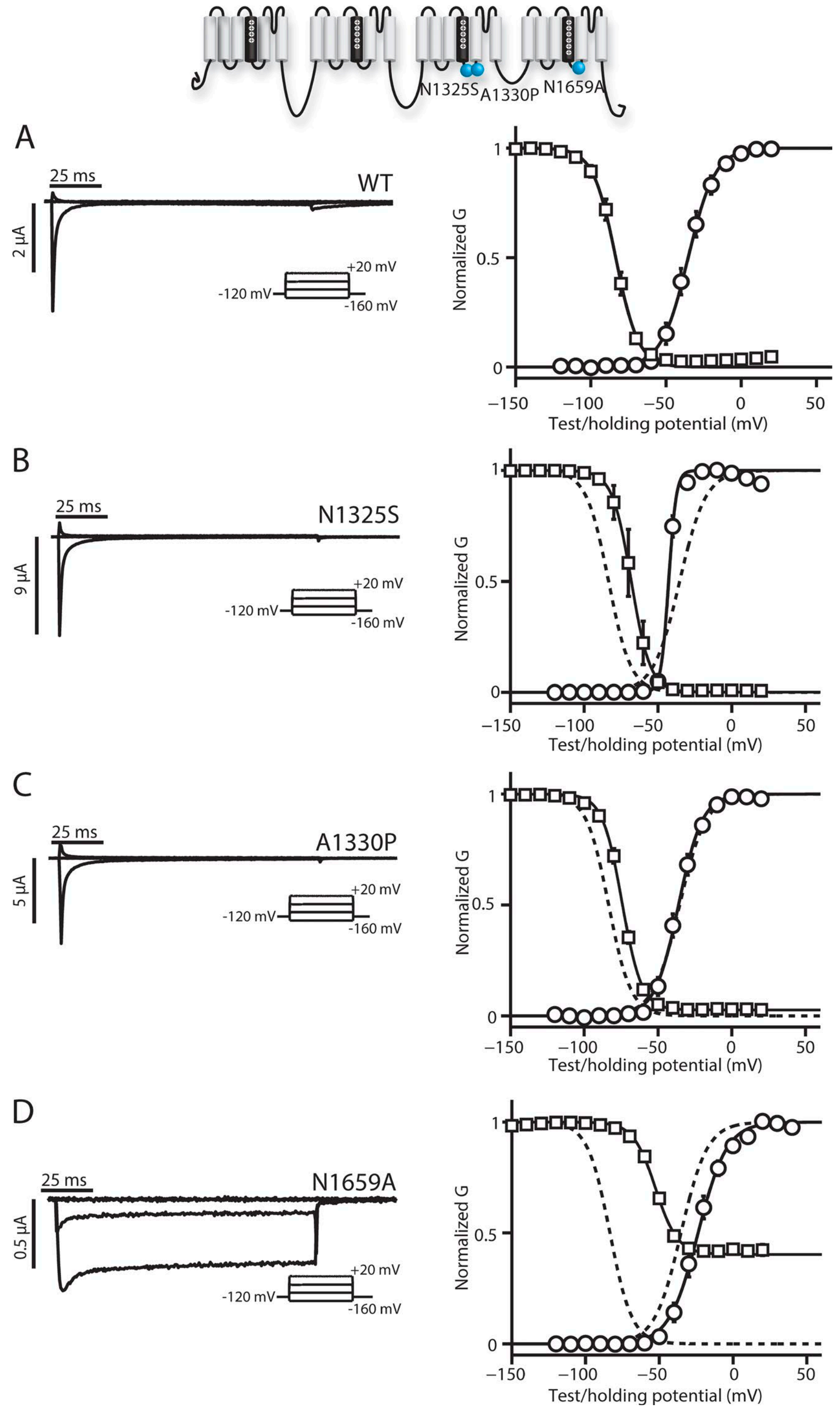

Figure S2. **Ionic currents and voltage dependence of steady-state parameters of WT and non-LFS mutant channels.** Na^+ currents were recorded from human $Na_V1.5$ channels carrying no fluorescence or background mutations. The mean ± SEM for five to seven cells is reported. Activation and inactivation curves were obtained via the protocols described in Fig. 1. (A–D) Traces and graphs were plotted for WT (A), N1325S (B), A1330P (C), and N1659A (D). (left) Ionic currents from channels were recorded during 200-ms-long depolarizing pulses ranging from −160 to 20 mV in 20-mV steps. Current traces corresponding to only −160, −100, −40, and 20 mV are shown for clarity. (right) Voltage dependence curves of steady-state activation (GV, black circles) and SSI (black squares) for each channel were created. Curves were constructed as described in the Materials and methods section. See Table 1 for Boltzmann fit parameters.
